# Rainfall and Temporal Influences on the Hydrodynamics of a Vertical Subsurface Flow Constructed Wetland Treating Domestic Sewage

**DOI:** 10.1002/wer.70324

**Published:** 2026-03-04

**Authors:** Galilleu Silva, Édio Damásio da Silva Júnior

**Affiliations:** ^1^ Goiano Federal Institute of Education, Science and Technology Rio Verde Brazil

**Keywords:** *Chrysopogon zizanioides*, clogging, evapotranspiration, hydrodynamic tracer

## Abstract

This study aimed to evaluate the hydrodynamic behavior of a vertical subsurface flow constructed wetland (VSSF‐CW) treating domestic sewage by applying a saline tracer, comparing system performance in operational Years 3 (NR‐3) and 5 (NR‐5), and assessing the influence of a rainfall event (R‐5). Electrical conductivity monitoring was used to construct residence time distribution (RTD) curves for all tests, enabling detailed characterization of hydraulic behavior. As a result, the system exhibited highly dispersed flow (*d* > 1.21; *N* < 2.07) with a tendency toward continuous stirred tank reactor (CSTR) behavior. A comparison between NR‐3 and NR‐5 tests revealed significant differences (*p* < 0.05, *t* test) in the hydrodynamic parameters. The rainfall event (R‐5) had a statistically significant effect (*p* < 0.05, *t* test), decreasing hydraulic retention time, increasing dilution, and enhancing dispersive flow within the treatment unit. These findings highlight the importance of long‐term hydrodynamic monitoring in VSSF‐CW systems and demonstrate how operational conditions and external factors such as rainfall can influence treatment performance.

## Summary

- VSSF‐CW operated with high dispersion, close to the CSTR model, but with significant deviations from ideality.
- The system exhibited preferential flows and hydraulic short circuits.
- The feeding regime induced daily oscillations, alternating advective transport (daytime) and diffusive transport (nighttime).
- Between Year 3 and Year 5, root growth and biofilm reduced dispersivity, whereas volumetric efficiency remained stable.
- Event R‐5 reduced HRT, altered dispersion parameters, and promoted dilution and flushing, increasing advective flow.

## Graphical abstract

The constructed wetland hydrodynamic study indicated the formation of preferential flow paths between Years 3 and 5. Rainfall reduced the retention time and increased dispersive flow in the sewage treatment unit.

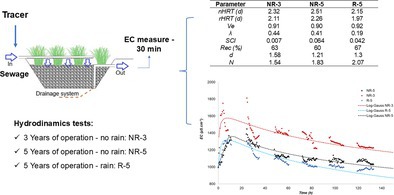

## 1 Introduction

Sewage treatment through constructed wetlands (CWs) has been consolidated since the 1950s, establishing itself as a sustainable and effective alternative for the removal of biodegradable pollutants, mainly through biological processes at the secondary and/or tertiary level, demonstrating great potential for the decentralized treatment of domestic, industrial, and agricultural effluents (Retta et al. 2023; Vymazal 2022).

CWs stand out for their high efficiency in removing organic matter, nitrogen, pathogenic microorganisms, and other pollutants from domestic sewage, while also representing a low‐cost technology with simple operation and maintenance requirements. Their strong landscape integration enhances social and environmental acceptance compared to conventional treatment systems (Dai et al. 2024; Liu et al. 2024; Zhang et al. 2024).

In many cases, the design and construction characteristics of CWs can result in less‐than‐ideal performance. Various factors directly influence treatment efficiency, including system geometry, the stage of vegetation development, and climatic factors such as evapotranspiration and environmental temperature (Kadlec and Wallace 2009; Liu et al. 2024).

Hydraulic performance stands out as a critical factor, as it conditions the hydraulic retention time (HRT), flow distribution, and the occurrence of undesirable phenomena such as dead zones, hydraulic short circuits, and clogging. These limitations reduce the useful volume of treatment cells and compromise pollutant removal. As CWs age, they may develop hydraulic constraints that reduce treatment efficiency, particularly due to clogging, the formation of stagnant regions, and the development of preferential pathways (Ioannidou and Pearson 2019; Miranda et al. 2019; Sabokrouhiyeh et al. 2017; Zhao et al. 2025).

Most CW projects disregard important components of the hydrological cycle, such as rainfall (*R*) and evapotranspiration (*ET*). *ET* directly influences the performance of these systems, as it modifies the transport of constituents through the porous medium, reducing volumetric flow and increasing the HRT. This prolongation of the HRT can favor pollutant removal processes and may also result in higher concentrations of dissolved constituents due to the reduction in water volume. Conversely, *R* acts in the opposite way: By increasing the water volume inside the CW, it promotes the dilution of the sewage and, consequently, reduces the HRT of the treatment environment, which can compromise the removal efficiency of some pollutants (Amiri et al. 2022; Beebe et al. 2014; Harne et al. 2023; Magalhães Filho et al. 2018; Silva Júnior and de Souza 2024). Therefore, both *ET* and *R* must be considered in the design and operation of CWs, as they represent key variables for hydraulic stability and the overall performance of the system.

To evaluate CW hydrodynamics, pulse‐tracer tests constitute a fundamental tool, as they allow for characterizing the flow hydrodynamics and identifying deviations from ideal hydraulic behavior (Du et al. 2024; Stephenson and Sheridan 2021).

Tracers, defined as chemical or biological substances capable of tracking water movement within the system, provide valuable information on parameters such as HRT, dispersion, and the presence of short circuits (Moraes et al. 2019; Stephenson et al. 2021).

Among the most applied are chemical tracers and fluorescent dyes (Kadlec and Wallace 2009), with sodium chloride (NaCl) standing out because of its low cost, high solubility, easy availability, and simple, accurate detection, making it a widely adopted option in hydrodynamic studies of CWs (Matos et al. 2017; Wang et al. 2023).

This study addresses a relevant gap in the scientific literature, as no previous studies have reported tracer‐based hydrodynamic evaluations of CWs conducted specifically during rainfall events, despite the known influence of precipitation on system hydraulics. Furthermore, only a limited number of studies have performed multi‐year hydrodynamic assessments, making longitudinal analyses of system maturation and flow behavior particularly scarce. In addition, tracer applications in full‐scale vertical subsurface flow CW (VSSF‐CW) systems remain limited in the literature, with most hydrodynamic investigations relying on pilot‐scale units. The present study therefore advances the field by combining full‐scale tracer testing, multi‐year evaluation, and rainfall‐driven hydrodynamic analysis within a single experimental framework.

In this context, the use of hydrodynamic tracer tests proves to be an indispensable tool for identifying deviations in flow, evaluating the influence of precipitation on the dilution and HRT of CWs, and understanding the cumulative effects of operating time on the system's hydraulic structure. Thus, the proposed objective (to evaluate the hydrodynamic behavior of a full‐scale VSSF‐CW as a function of rainfall and operating time) is justified by the need to integrate climatic and operational factors into the understanding of these systems' performance, providing support for their design, management, and optimization.

## 2 Materials and Methods

### 2.1 Domestic Sewage Treatment

The domestic sewage treatment system was implemented at the Goiano Federal Institute of Education, Science and Technology, in the city of Rio Verde, Brazil, in July 2016, at the geographical coordinates 17°48′20″ S and 50°54′24″ W. Qualitative information on sewage treated in this system is available in Santos et al. (2024) and Silva Júnior and de Souza (2024).

**FIGURE 1:**
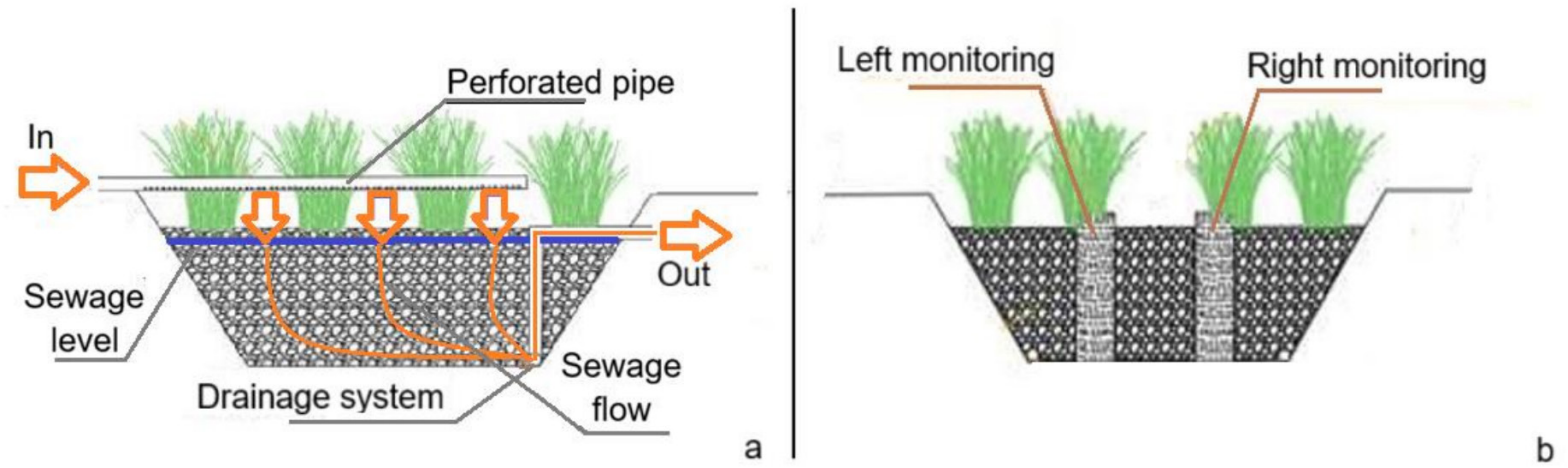
Longitudinal (a) and cross‐sectional (b) profiles of the studied VSSF‐CW.

It is a decentralized domestic sewage treatment system, originating from five residences of the mentioned institution, with an initial design flow rate of 1200 L·day^−1^. The treatment system consists, in sequence, of a septic tank, a suction tank, a distribution tank (or passing chamber), and a VSSF‐CW (Figure 1).

The preliminary treatment consists of a septic tank (2.2 × 1.2 × 1.2 m), designed for the retention of settleable and coarse solids. Following the septic tank is a suction tank, with a useful volumetric capacity of 500 L. Subsequently, the sewage accumulated in this tank is pumped to a distribution tank (1000 L), from where it flows, by gravity, to the VSSF‐CW.

VSSF‐CW operates under an intermittent (batch) feeding regime. As sewage is generated by the households, it first flows into the septic tank and then into the suction tank. When the liquid level in the suction tank reaches the upper threshold, a pump controlled by a float switch is activated and delivers sewage to the wetland until the level drops to the lower threshold. This cycle is repeated according to the pattern of sewage generation in the residences. Such intermittent loading results in periodic variations in inflow.

The VSSF‐CW has an inverted truncated pyramid shape, with a nominal nHRT of nearly 2 days, dimensions of 2.8 m in length and 2.8 m in width (at the average bed depth), 0.85 m in depth, 0.20 m of freeboard, and slopes inclined at 60°. The structure was excavated in the soil and waterproofed with a 1.5‐mm‐thick high‐density polyethylene geomembrane. The support medium consists of #2 crushed stone (gravel), and the bed was vegetated with vetiver grass (*Chrysopogon zizanioides*).

The nHRT was obtained by dividing the effective volume of the VSSF‐CW (2.35 m^3^) by the average daily inflow (1.2 m^3^·day^−1^), which was measured over the 3 years preceding the first tracer test. The effective volume was calculated considering the geometry of the unit (an inverted truncated pyramid) and the porosity of the gravel medium (#2 gravel, 55% porosity).

To measure the inflow and outflow rates of the VSSF‐CW, high‐precision volumetric water meters, model Saga ¾″ R315, were used. The water meter installed at the CW inlet has a bypass system, occasionally activated for equipment cleaning.

The sewage was applied to the VSSF‐CW through a single, longitudinally perforated polyvinyl chloride pipe. The wetland operated under saturated flow despite the intermittent feeding regime. The outlet pipe was positioned 0.85 m above the bottom of the unit, ensuring a permanently saturated zone of 0.85 m.

### 2.2 Hydrodynamic Evaluation

**FIGURE 2:**
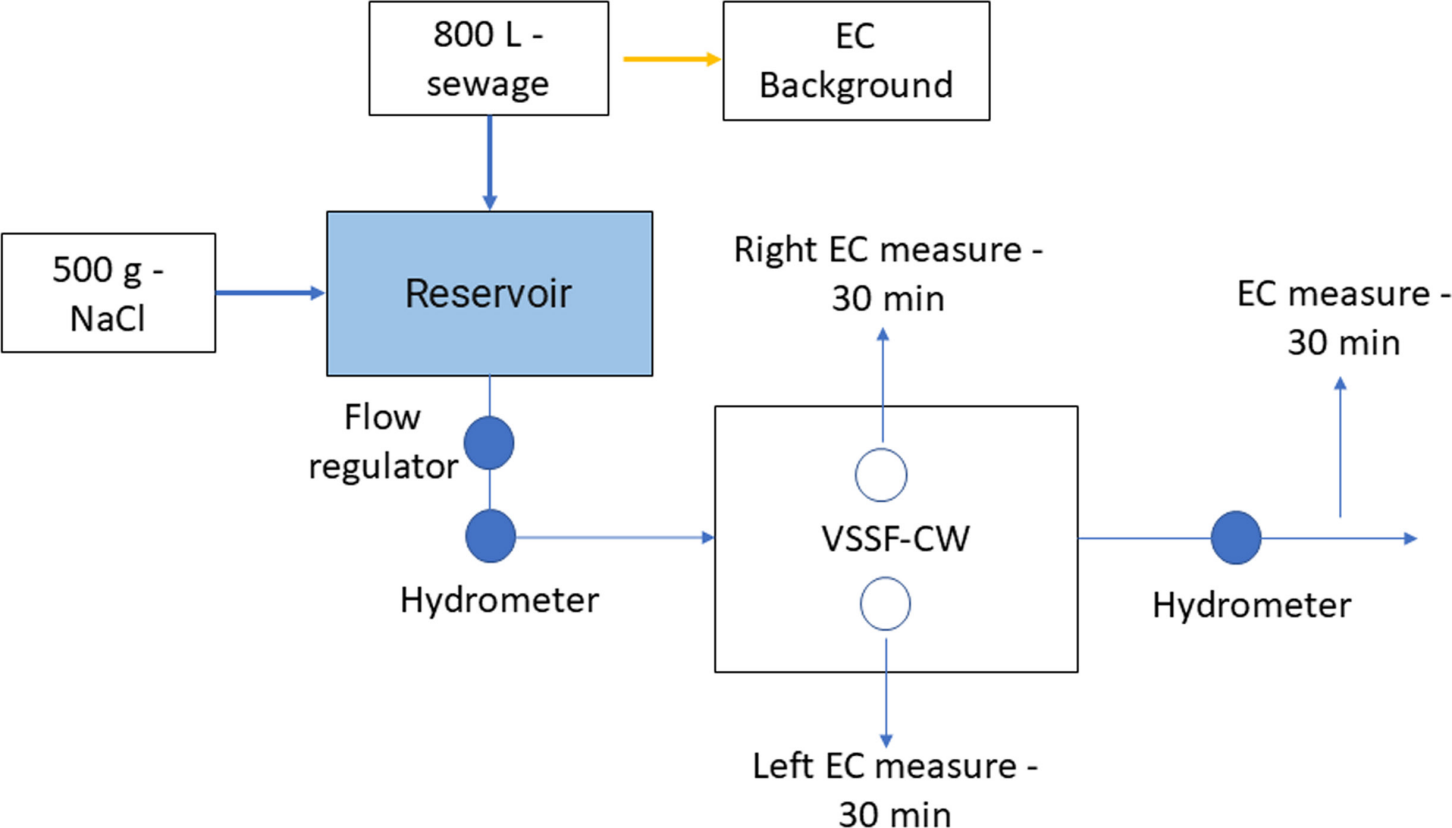
Hydrodynamic evaluation procedures in the VSSF‐CW using the pulse‐tracer method.

The evaluation of the hydrodynamic behavior of the VSSF‐CW as a function of the variables “time” and “rainfall” followed the pulse‐tracer method, as described in Figure 2. Three tests were conducted using sodium chloride (NaCl) as the saline tracer, monitored via electrical conductivity (EC): a nonrain event test, after 3 years of operation (NR‐3); a rain event test, after 5 years of operation (R‐5); and a nonrain event test, after 5 years of operation (NR‐5).

The R‐5 test was conducted during a rainfall event with a total accumulation of 50 mm over 2 days (Days 2 and 3 of the experiment). Weather forecasts were monitored to ensure that the hydrodynamic test would coincide with rainy conditions. In contrast, the NR‐3 and NR‐5 tests were performed during dry‐season periods, allowing a direct comparison of hydrodynamic behavior under distinct climatic conditions. It is important to note that the central region of Brazil has a well‐defined rainfall regime, with approximately 6 months of intense precipitation followed by 6 months of pronounced dry weather, which facilitated the planning and execution of tests under contrasting hydrological scenarios.

The use of NaCl was adopted because of its low acquisition cost and the possibility of relating EC to NaCl concentration, yielding a standard curve. To quantify the EC in the sewage, an Akso AK88 probe was used, with a detection range of 0–2000 μS·cm^−1^.

The methodology employed for the hydrodynamic tracer tests was recommended by Kadlec and Wallace (2009). According to the authors, the first step after selecting the tracer compound consists of defining the tracer's background concentration in the medium. This concentration was determined from 10 EC measurements, carried out at 1‐h intervals, of the domestic sewage before the pulse application. Background conductivity was measured to ensure that only the conductivity attributable to the added NaCl was considered in the mass balance. Potential ions released by vegetation or the substrate during the short duration of the tracer test are not expected to meaningfully affect conductivity and are generally regarded as negligible in tracer‐based hydrodynamic assessments.

An amount of 500 g of NaCl was diluted by manual mixing in 800 L of sewage in the reservoir, adjusting the EC value to approximately 2000 μS·cm^−1^. The selected NaCl mass ensured a clear and easily detectable EC signal above background variability while remaining sufficiently low to avoid density‐driven effects that could distort flow patterns. Subsequently, the valve was opened, and the sewage (with the tracer) was released into the VSSF‐CW. In this way, the flow feed occurred as a pulse.

To minimize the phenomenon of density stratification, Kadlec and Wallace (2009) recommend that the application time (pulse) not exceed 10% of the nominal HRT (nHRT) of the treatment cell. According to preliminary tests, the application time for 800 L of sewage in the VSSF‐CW was approximately 3 h, which corresponds to about 6% of the cell's nHRT. After the pulse application, the sewage EC was monitored at seven sampling points: inside the treatment cell (at depths of 20, 40, and 80 cm, in perforated pipes installed on the right and left sides of the treatment unit) and at the VSSF‐CW outlet, as per Figures 1 and 2.

Monitoring was carried out at 30‐min intervals (from 7:00 a.m. to 7:00 p.m.) for six consecutive days, until the EC of the sewage at the VSSF‐CW outlet returned to the background value.

The following hydrodynamic behavior estimators were calculated: real HRT (*rHRT*)—Equation (1), volumetric efficiency (*V*_*e*_)—Equation (2), hydraulic efficiency (*λ*)—Equation (3), short‐circuiting index (*SCI*)—Equation (4), and tracer recovery percentage (*Rec*)—Equation (5).

(1) $$\text{rHRT}=\frac{\int t\cdot c\left(t\right)\cdot \mathrm{dt}}{\int c\left(t\right)\cdot \mathrm{dt}}$$

(2) $$V_{e}=\frac{\text{rHRT}}{\text{nHRT}}$$

(3) $$\lambda =\frac{t_{p}}{\text{nHRT}}$$

(4) $$\mathrm{SCI}=\frac{t_{i}}{\text{rHRT}}$$

(5) $$\mathrm{Rec}=\frac{\sum \left[\text{NaCl}\right]\cdot Q\cdot t_{m}}{M}$$

where *nHRT* is the nominal HRT (days), *t* is the time of measured concentration (days), *c*(*t*) is the NaCl concentration at time *t* (mg·L^−1^), *t*_*p*_ is the time corresponding to the peak concentration (days), *t*_*i*_ is the time of first tracer appearance (days), *Q* is the sewage flow rate (m^3^·s^−1^), *t*_*m*_ is the data acquisition interval (days), and *M* is the mass of tracer added (g).

The EC values obtained were adjusted by subtracting the background concentration for each test to normalize the data and significantly represent the tracer concentration, enabling the generation of the residence time distribution (RTD) curves in the VSSF‐CW, considering the idealized flow pattern. The curves were elaborated from the monitoring of the NaCl concentration as a function of time, according to Equation (6), as suggested by Kadlec and Wallace (2009).

(6) $$\text{RTDi}=\frac{\mathrm{Ci}\cdot Q}{M}$$

where *RTDi* is the curve point at time *t*_*i*_ and *Ci* is the NaCl concentration at time *t*_*i*_ (mg·L^−1^).

The conversion of EC to NaCl concentration (*Ci*) was based on a laboratory‐derived linear regression between EC and known NaCl masses. This calibration step is standard practice in salt‐tracer hydrodynamic studies, as it enables quantification of the tracer mass recovered at the outlet and, consequently, calculation of the recovery rate (*Rec*).

Equation (6) generated several points, correlating *RTDi* and time *t*_*i*_, generating the overall RTD curve. To generate the representative RTD equation, the GraphPad Prism 7 software was used.

Considering the axial dispersion model, the spreading of the pulse throughout the reactors was measured by means of the dispersion coefficient *D* (m^2^·s^−1^). According to Levenspiel (2000), Equation (7) is a dimensionless group that characterizes the spreading of the flow in the reactor, defining the dispersion number *d*.

(7) $$d=\frac{D}{u\cdot L}$$

where *u* is the average flow velocity (m·s^−1^) and *L* is the reactor length (m).

The result obtained from Equation (7) indicates the dispersion number of the VSSF‐CW and its proximity to an idealized model (continuous stirred tank reactor [CSTR] or plug‐flow reactor [PFR]). However, to obtain *d*, Levenspiel (2000) suggests using Equation (8), considering the variance (*σ*) of the data obtained.

(8) $$\frac{\sigma^{2}}{{\text{rHRT}}^{2}}=2\cdot \frac{D}{u\cdot L}$$

Equation (8) is valid only under a specific boundary condition of the axial dispersion model. This formulation corresponds to open–open boundary conditions, in which both the inlet and outlet behave as open boundaries with convective flow and no back‐mixing imposed at the reactor ends. These assumptions allow the variance of the RTD to be related directly to the dispersion number. The open–open boundary assumption idealizes the hydraulic behavior at the inlet and outlet and may not fully represent systems with complex inlet structures, dead zones, or partial short‐circuiting (Levenspiel 2000).

For *d* values < 0.01, there is a tendency for the sewage flow through the treatment cell to approximate the PFR model. On the other hand, if *d* > 0.01, it indicates a significant deviation from PFR flow. The tank‐in‐series (TIS) model was used to estimate the number of tanks (*N*) that represent the VSSF‐CW, according to Equation (9) (Levenspiel 2000).

(9) $$\sigma_{ϴ}^{2}=\frac{1}{N}$$

where *σ*_*ϴ*_ is the dimensionless variance of the tracer pulse, which can be calculated using Equation (10)

(10) $$\sigma_{ϴ}^{2}=\frac{\text{rHRT}-t_{p}}{\text{rHRT}}$$

where *t*_*p*_ is the time corresponding to the peak concentration (*d*).

If the value of *N* is close to 1, it indicates that the flow pattern is like a CSTR, whereas *N* → ∞ indicates PFR‐type flow (Levenspiel 2000).

### 2.3 System Water Balance

The water flow measured in the VSSF‐CW considered the influent sewage volume (*IS*), the effluent sewage volume (*ES*), the precipitation (*P*), and the evapotranspiration (*ET*) in the treatment unit.

The sewage inflow and outflow volumes of the CW were quantified using installed high‐precision volumetric water meters, model Saga ¾″ R315. The volume of rainwater that entered the system was calculated based on the value measured by a rain gauge (Incoterm 4760 model) installed on site.

The daily *ET* was calculated during the tracer tests using Equation (11), based on the principle of mass conservation in a dynamic state.

(11) $$\mathrm{ET}=\text{IS}–\mathrm{ES}+R\pm \frac{\Delta H\cdot A\cdot \alpha }{1000}$$

where *ET* is the evapotranspiration (L·day^−1^), *IS* is the influent sewage (L·day^−1^), *ES* is the effluent sewage (L·day^−1^), *R* is the rainfall (L·day^−1^), *α* is the support medium porosity (%), *A* is the CW surface area (m^2^), and Δ*H* is the daily variation of liquid depth in the CW (m).

The sewage level in the VSSF‐CW was considered constant; thus, Δ*H* = 0 was adopted, because no significant changes were observed in the sewage level of the cell treatment over time.

### 2.4 Statistical Analysis

A statistical evaluation was carried out to identify the best nonlinear regression model, with the aim of generating curves capable of relating to the EC as a function of time *t*, thus promoting the normalization of the RTD curves. The GraphPad Prism 7.0 software was used for this purpose.

The model that presented the best initial fit was the sum of two Gaussian curves; however, after the normalization and differentiation of the observed values by the background concentration, the log‐Gaussian curve obtained the highest coefficient of determination (*R* ^2^) and the best fit.

To assess whether the differences observed among the hydrodynamic parameters were statistically significant, a Student's *t* test was applied with a 95% confidence level. This analysis was used to compare the NR‐3, NR‐5, and R‐5 events, allowing us to quantify the influence of rainfall and system maturation on the hydrodynamic response.

## 3 Results and Discussion

### 3.1 Water Balance

**FIGURE 3:**
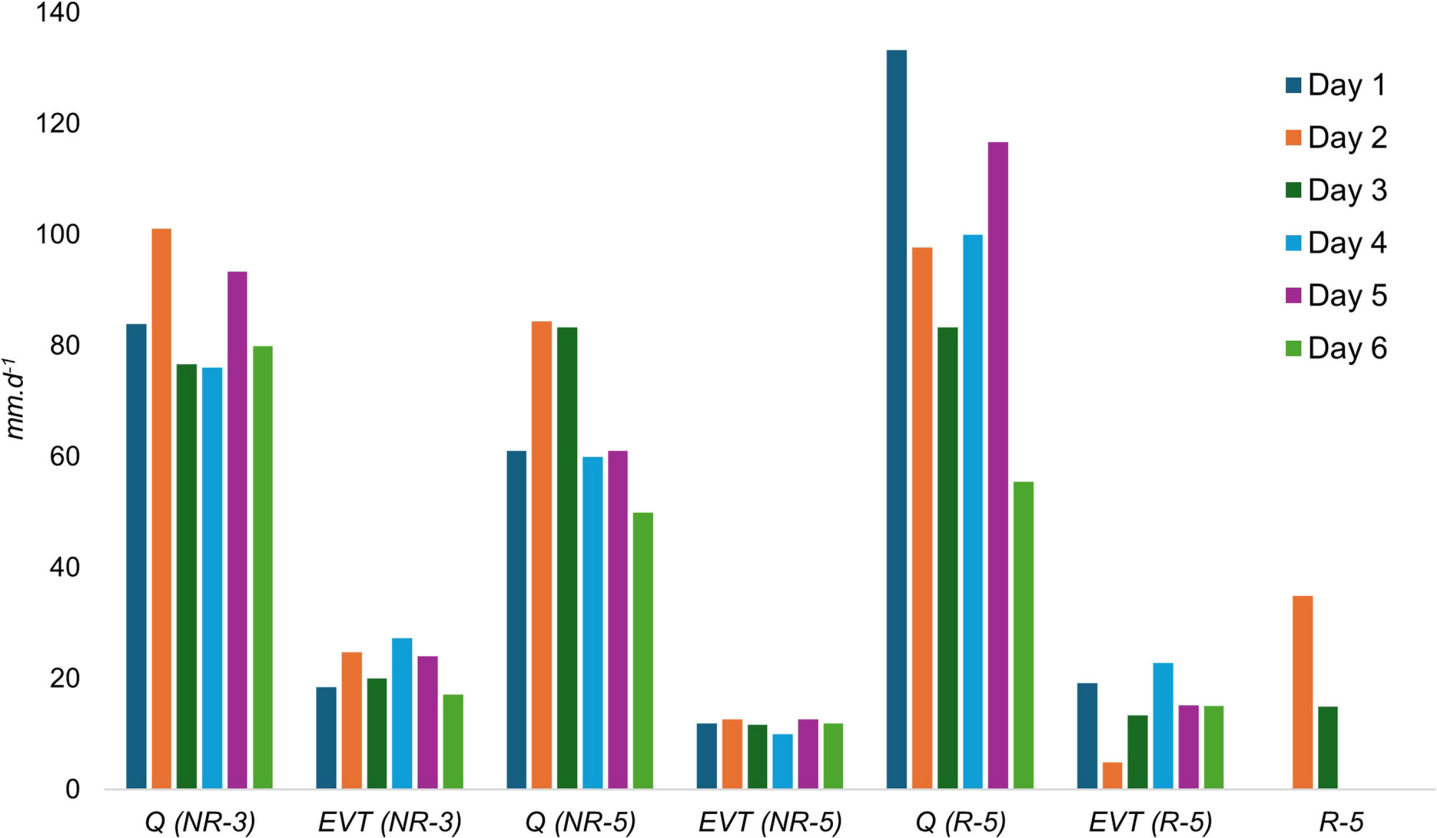
Water balance of the studied VSSF‐CW, considering *ET* and *R*, during the hydrodynamic tracer tests.

The water balance in CWs must consider water inputs, such as *R*, and outputs, such as *ET*, in addition to the sewage flow under treatment. *R* and *ET* are relevant variables that can influence pollutant removal efficiency in CWs, because precipitation may reduce pollutant concentration in the liquid medium, whereas evapotranspiration tends to increase it (Amiri et al. 2022; Harne et al. 2023; Magalhães Filho et al. 2018). Figure 3 shows the water behavior of the evaluated sewage treatment unit, considering *ET* and *R* data during the hydrodynamic tracer test.

During the hydrodynamic tests, the influent sewage flows in VSSF‐CW exhibited high variability, with the following average values: *Q* (NR‐3) = 85.2 ± 10.0 mm·d^−1^, *Q* (NR‐5) = 66.7 ± 13.9 mm·d^−1^, and *Q* (R‐5) = 97.8 ± 26.6 mm·d^−1^. Such variations are common in domestic sewage systems, as they result from factors such as residents' daily water consumption habits and local climatic conditions (mainly precipitation and temperature) (Tchobanoglous et al. 2014).

Only in the third hydrodynamic test (R‐5) did rainfall occur, between Days 2 and 3, with approximately 468 L of rainwater, or 50 mm of precipitation, entering the VSSF‐CW. This value corresponded to about 50% of the average daily influent sewage flow in the treatment unit evaluated during period R‐5.

In theory, excessive amounts of rainfall can impact the hydrodynamics of sewage treatment systems. This means that the concentration of contaminants (such as organic matter or nutrients) present in the influent sewage may be reduced even before treatment processes take place. However, this same additional water volume, which causes dilution, also significantly increases the total hydraulic load in the system, thereby reducing its HRT.

The estimated average daily *ET* also showed variations during the hydrodynamic tests: *ET* (NR‐3) = 22.0 ± 3.9 mm·d^−1^, *ET* (NR‐5) = 11.8 ± 1.0 mm·d^−1^, and *ET* (R‐5) = 15.1 ± 6.1 mm·d^−1^. *ET* can be divided into evaporation of water directly exposed to the air and plant transpiration, driven by the transformation of solar energy into latent heat of vaporization of liquid water. It depends on meteorological factors such as temperature, air humidity, wind speed, and solar radiation (Chen et al. 2022; Ghiat et al. 2021).

The average daily losses due to *ET*, calculated in relation to system inputs (*Q* + *R*), were 25.8%, 17.7%, and 10.2% for tests NR‐3, NR‐5, and R‐5, respectively. In addition to meteorological factors, the age and development stage of vegetation also influence the *ET* rate (Chen et al. 2022; Ghiat et al. 2021). The 2‐year interval between tests NR‐3 and NR‐5, for example, likely altered the growth stage of the vetiver grass species, which may explain part of the variation observed.

Significant percentage variations in *ET* in CWs are widely reported in the literature, depending on climatic combinations and the plant species used. It is possible to design CW‐based treatment systems with “zero discharge,” in which *ET* is the only water output pathway from the system (Amiri et al. 2022; Beebe et al. 2014; Frédette et al. 2022; Harne et al. 2023; Nivala et al. 2022; Reis et al. 2023, 2024; Santos et al. 2024).

### 3.2 Hydrodynamic Evaluation

**FIGURE 4:**
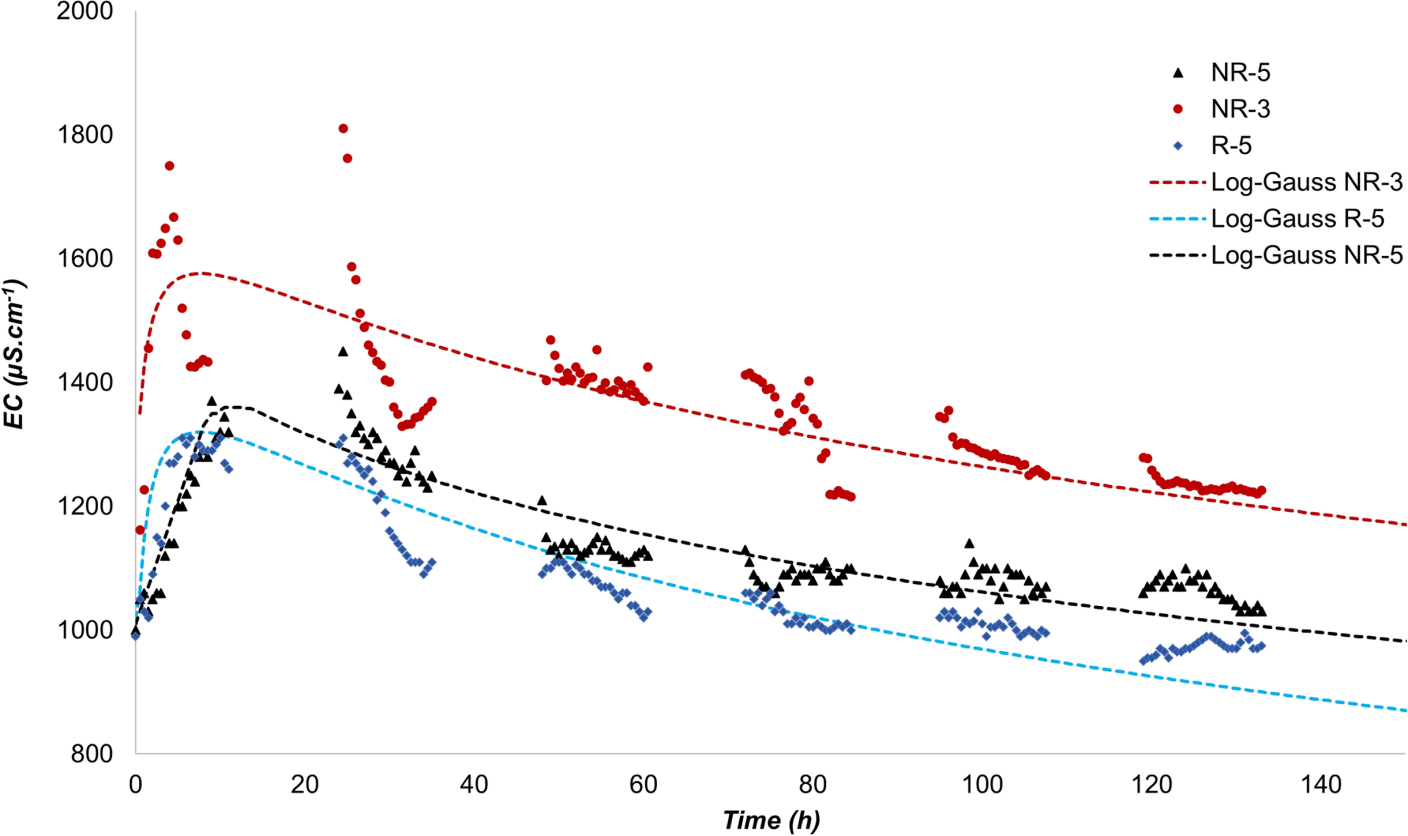
Distribution curves of the VSSF‐CW from the hydrodynamic tracer tests performed.

The results of the hydrodynamic test with saline tracer (NaCl) in the VSSF‐CW, presented as an RTD curve, are shown in Figure 4.

The log‐Gaussian model provided the best statistical fit (*R* ^2^ > 0.79) and accurately represented the behavior of the saline tracer in the studied VSSF‐CW. This distribution is widely applied in hydrodynamic analyses of nonideal reactors because its characteristic skewness is consistent with the behavior predicted by the axial dispersion model, and its parameters can be related to the dispersion number (Kadlec and Wallace 2009).

As shown in Figure 4, a log‐Gaussian (log‐normal) RTD curve typically displays a rapid rise in tracer concentration at early times (first 10 h), reflecting the arrival of the fastest flow paths through the porous medium, followed by a progressively slower, long‐tailed decline as the tracer moves through regions with lower velocities or greater dispersion. This asymmetric shape, characterized by a sharp leading edge and an extended trailing tail, is a hallmark of systems governed by axial dispersion, where part of the tracer pulse is transported quickly whereas another fraction is retained longer within the pore structure (Bodin et al. 2012).

Such a result is consistent with the design characteristics of the VSSF‐CW, specifically its geometry (inverted pyramid trunk shape) and the downflow feeding system, which promote rapid tracer dispersion (Kadlec and Wallace 2009). The same result has been observed in other studies applying pulse tracers in VSSF‐CW sewage treatment units (Du et al. 2024; Moraes et al. 2019; Wang et al. 2023).

A log‐Gaussian RTD curve of this shape indicates high dispersion (*d*), reflected by its pronounced asymmetry and long trailing tail. Such behavior is characteristic of systems in which axial dispersion is significant, causing a broad distribution of residence times. As dispersion increases, the RTD progressively departs from the plug‐flow ideal and moves toward the hydrodynamic behavior associated with a CSTR‐like regime (Kadlec and Wallace 2009; Levenspiel 2000).

The use of log‐Gaussian RTD fitting is supported by classical theoretical treatments of dispersion in porous media and packed‐bed reactors, as well as by applications in CWs, where skewed RTD responses are commonly observed (Bodin et al. 2012; Brovelli et al. 2011; Dittrich et al. 2021).

The NR‐3 test recorded a higher peak of absolute electrical conductivity compared to the NR‐5 and R‐5 tests. This difference is directly attributed to the baseline (background) of the influent, which showed a higher background conductivity in this test (1157 μS·cm^−1^) compared to the others (≈1050 μS·cm^−1^). It is important to distinguish this increase in the absolute measurement value (caused by the sewage matrix) from the hydrodynamic behavior of the reactor. Such variations in influent sewage quality are expected and may be associated with seasonal, climatic factors or the habits of the local population (Tchobanoglous et al. 2014).

The intermittent (batch) feeding regime of the treatment unit induced periodic fluctuations in tracer concentration. It was systematically observed that concentrations measured at the beginning of the daytime cycle were higher than those recorded at the end of the previous cycle. This pattern is interpreted as a reflection of the transition between the dominant mass‐transport mechanisms: advection (during flow periods) and molecular diffusion (during nighttime stagnation periods).

Advection is the main mass‐transport mechanism in porous media, carrying solutes through the bed at the average fluid velocity and determining the movement of the contaminant plume. However, because water follows tortuous paths around solid particles, pore‐scale velocities are non‐uniform, which leads to hydrodynamic dispersion, the spreading of the tracer as an inherent consequence of advective transport (Shih and Wang 2021; Wehbe et al. 2024; Yuan et al. 2023).

Molecular diffusion becomes the dominant transport mechanism in the absence of advective flow, such as under stagnant nighttime conditions. Driven by the random Brownian motion of molecules, it promotes the migration of solutes from regions of higher to lower concentration (Berg et al. 2022; Wehbe et al. 2024). As highlighted by Gaullier et al. (2020), this process governs solute movement when flow is absent and leads to the slow homogenization of contaminants within VSSF‐CWs.

In addition to advection and molecular diffusion, density stratification (saline stratification) may have been a critical hydrodynamic factor. The introduction of an influent with higher salinity, and consequently higher density, can induce vertical segregation in the VSSF‐CW (Kadlec and Wallace 2009). The denser sewage tends to flow preferentially along the bottom of the treatment unit. Considering that the effluent outlet structure is located precisely in this region (bottom), this phenomenon may create a preferential flow path, characterizing a hydrodynamic short circuit.

Vegetation can also exert a significant hydrodynamic effect in VSSF‐CWs by increasing resistance to sewage flow. This resistance, however, is inherently heterogeneous because of the irregular distribution of stems and root systems. This complex interaction can alter the flow regime, induce turbulence and convective currents, and modify the flow path, promoting the formation of preferential pathways (hydraulic short circuits) and stagnation zones (dead zones) (Jin et al. 2023; Matos et al. 2017, 2018; Miranda et al. 2019).

The evaluation of the effect of precipitation (test R‐5) confirmed a statistically significant difference (*p* < 0.05, *t* test) compared to NR‐5. An addition of about 50% rainwater was measured in the VSSF‐CW on Days 2–3. The 50‐mm precipitation during this period may have been sufficient to significantly alter the hydrodynamics of the studied VSSF‐CW. In addition, the average sewage flow applied in R‐5 was 68% higher than in NR‐5, which makes it difficult to establish whether the observed difference between the tests was solely due to rainfall. Nevertheless, a direct effect of rainfall was observed: As shown in Figure 4, there was a clear decay in EC during the event (Hours 30–40), which strengthens the hypothesis of momentary dilution of sewage by rainwater.

Precipitation can drastically alter the hydrodynamics of a CW, primarily by introducing a pulse of water volume. This sudden increase in total flow (sewage + rainfall) raises the advective velocity of the fluid, leading to a reduction in HRT. The most critical hydrodynamic consequence is the induction of hydraulic short circuits, where water (both rainwater and sewage) passes through the unit without sufficient contact time with the filter medium and biofilms, compromising treatment removal efficiency. In addition to altering volume and HRT, rainfall introduces a dilution effect. By adding water with low solute concentration, it reduces the final pollutant concentration in the effluent, an effect that may mask the decline in hydrodynamic removal efficiency.

**FIGURE 5:**
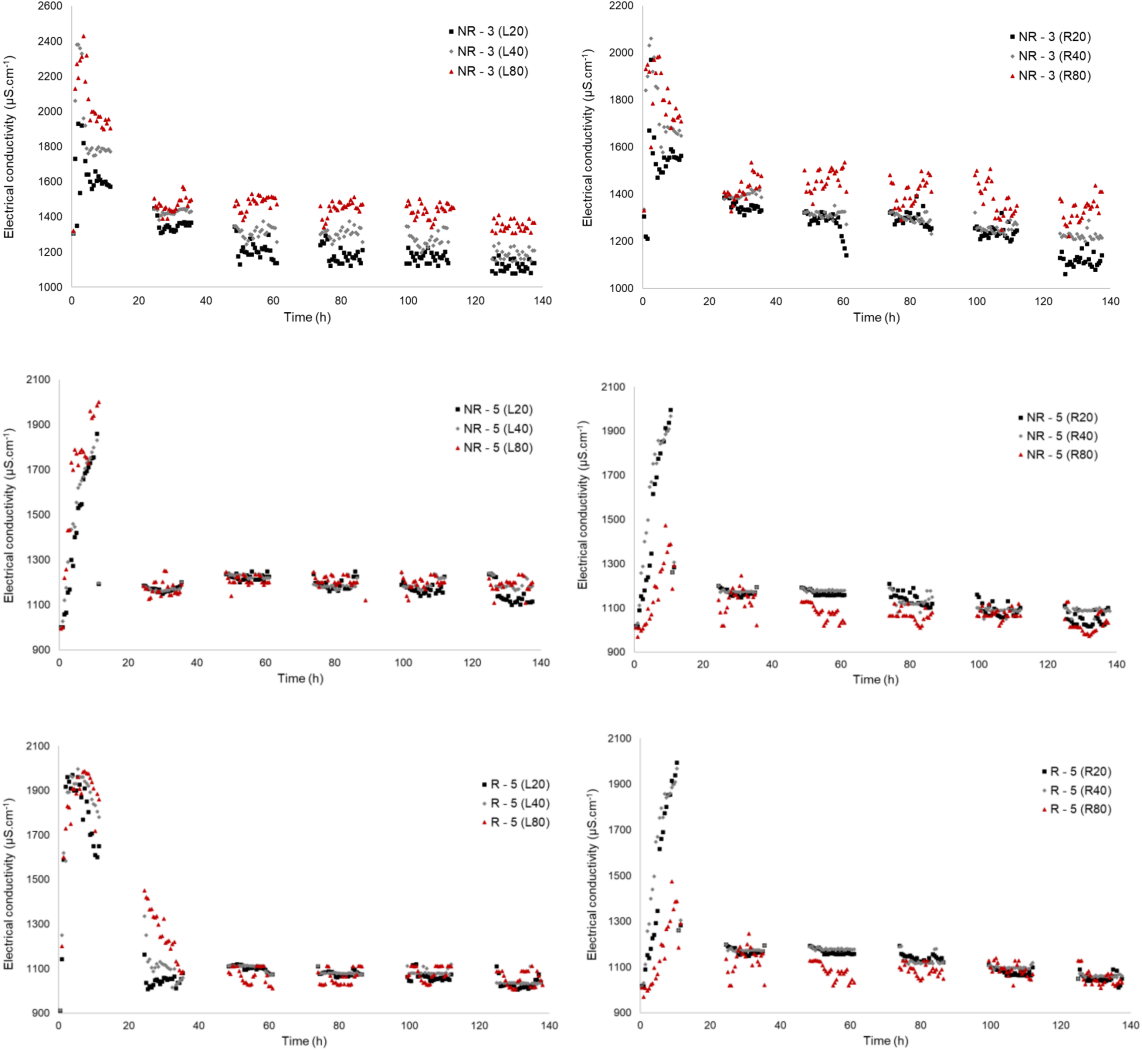
Hydrodynamic behavior of the tracer as a function of depth (20, 40, and 80 cm) at the left (L) and right (R) measurement points of the VSSF‐CW in the tests performed.

Figure 5 compares tracer hydrodynamic behavior as a function of depth (20, 40, and 80 cm) at the left (L) and right (R) measurement points of the VSSF‐CW in the tests performed.

The tests revealed asymmetric hydrodynamic distribution in the VSSF‐CW, characterized by a preferential flow of the tracer (sewage) through the left side of the unit. The magnitude of this asymmetry, quantified by the average difference in EC between L and R sides, was more pronounced in Year 3 (difference of 24%). A reduction in this phenomenon was observed in Year 5, when the average EC difference between the sides decreased to 16%. This asymmetry in dispersion is a strong indication of the formation of “preferential pathways” and “dead zones,” phenomena that reduce the effective contact time and, consequently, the pollutant removal efficiency (Jin et al. 2023; Matos et al. 2018; Miranda et al. 2019). The probable cause is related to constructive or operational aspects, such as a failure in the inlet piping that directs more sewage to the left side (or a partial obstruction on the right side, promoted by greater growth of the planted vegetation's root system).

A clear reduction in saline stratification was also observed when comparing test NR‐3 with tests NR‐5 and R‐5. In Year 3, the system exhibited a distinct stratification behavior, evidenced by a vertical gradient in EC as a function of depth. In contrast, the Year 5 tests showed a tendency toward homogeneity in the EC profile. It is hypothesized that this alteration in hydrodynamic flow is associated with the maturation of the vetiver grass root system, which has a high growth rate (Mahmoudpour et al. 2021). Over the years, the growth and deepening of the roots, which can reach meters, likely altered the flow patterns, inducing greater vertical mixing and thus mitigating density‐driven segregation (Ioannidou and Pearson 2019).

A statistical comparison between NR‐3 and NR‐5 tests revealed significant differences (*p* < 0.05, *t* test) in the hydrodynamic parameters of the system. These results indicate that the wetland underwent measurable changes in its hydraulic behavior over the years, reflecting the effects of system maturation, root development, and structural evolution of the porous medium. The observed differences confirm that long‐term operation influenced the internal flow dynamics of the VSSF‐CW.

Table 1 presents the results of the parameters used to evaluate the hydrodynamic flow characteristics in the VSSF‐CW during the tests performed.

**TABLE 1:** Hydrodynamic parameters calculated from the tests in the VSSF‐CW.

Parameter	NR‐3	NR‐5	R‐5
*nHRT* (days)	2.32	2.51	2.15
*rHRT* (days)	2.11	2.26	1.97
*V*_*e*_	0.91	0.90	0.92
*λ*	0.44	0.41	0.19
*SCI*	0.007	0.064	0.042
*Rec* (%)	63	60	67
*d*	1.58	1.21	1.3
*N*	1.54	1.83	2.07

*Note: nHRT* is the nominal hydraulic retention time, *rHRT* is the real hydraulic retention time, *V*_*e*_ is the volumetric efficiency, *λ* is the hydraulic efficiency, *SCI* is the short‐circuiting index, *Rec* is the tracer recovery rate, *d* is the dispersion number, and *N* is the number of tank‐in‐series (TIS) model.

The R‐5 test presented the highest total inflow volume, combining sewage flow and precipitation, consequently resulting in the lowest nHRT and rHRT values. This is an expected result because HRT is inversely proportional to inflow rate, demonstrating that higher flows induce shorter HRTs, generating an advective flow regime (Ioannidou and Pearson 2019; Yuan et al. 2023).

rHRT provides a more accurate metric of the system, as it is calculated from experimental tracer data. It reflects the real internal hydrodynamics, incorporating flow deviations such as short circuits and dead zones (in addition to external factors, such as precipitation in the R‐5 test) (Kadlec and Wallace 2009). On average, rHRT was about 10% lower than nHRT, indicating that the volumetric efficiency (*V*_*e*_) of the reactor is approximately 0.90, meaning the volume effectively used by the flow is smaller than the total volume. rHRT tends to progressively diverge from the nominal (theoretical) value to the degree of clogging advances (Miranda et al. 2019; Ramos et al. 2022).

Hydrodynamics directly influences rHRT, the contact time between microorganisms and pollutants, and the degree of interaction between degraders (organic matter and nitrogen‐removing bacteria) and wastewater constituents. Effective removal of organic matter and nutrients requires sufficient time and contact for biochemical reactions to occur. Over the years, this interaction can be altered by hydraulic issues such as short‐circuiting, dead zones, or preferential pathways, which reduce treatment reliability (Tchobanoglous et al. 2014).

A significant evolution in SCI was observed when comparing the Year 3 and Year 5 tests. The SCI is an indicator of the flow regime, where values close to 1 tend to express PFR behavior, whereas values close to 0 indicate a tendency toward complete mix reactor (CSTR) behavior (Kadlec and Wallace 2009). In the present study, the tests conducted in Year 5 showed SCI values higher than those in Year 3.

The increase in hydraulic efficiency over the years is consistent with the observations of Matos et al. (2018), who reported changes in SCI associated with vegetation growth stages. It is estimated that the maturation of the root system and the development of biofilm in the filter medium were responsible for the increase in SCI. It is suggested that, although hydrodynamic deviations exist, clogging has not yet reached a critical level capable of causing a progressive deterioration of efficiency. Such indicators are important for the design of future VSSF‐CWs, which should consider this initial reduction in effective volume and monitor its progression over time.

Hydraulic efficiency (*λ*) is a key indicator of the degree of dispersion in VSSF‐CWs. Low *λ* values indicate behavior close to that of a completely mixed reactor (CSTR), whereas values near 1 approximate PFR behavior (Kadlec and Wallace 2009). In dry‐weather tests, the VSSF‐CW showed *λ* = 0.44 (NR‐3) and *λ* = 0.41 (NR‐5), confirming dispersed hydrodynamics with a clear tendency toward the CSTR model. However, during the R‐5 test (with rainfall), a drastic change was observed, with *λ* dropping to 0.19. This reduction, which indicates a sharp increase in dispersion, is possibly associated with the higher inflow rate (sewage + rainfall). This intense flow may have induced greater turbulence, resulting in the damping (flattening and spreading) of the tracer peak. Such evidence confirms the well‐known trade‐off of rainfall events: Although precipitation can dilute pollutants, it also reduces rHRT and may fundamentally alter the system's mixing regime.

The tracer recovery values (*Rec*) were considered consistent with those reported in the literature (generally in the range of 50%–70%), validating the hydrodynamic evaluation (Matos et al. 2017; Wang et al. 2023). The R‐5 test (with rainfall) stood out for presenting the highest mass recovery rate (67%). This result, although seemingly contradictory considering the dilution (concentration reduction) caused by rainfall, is technically expected: The additional rainfall volume acted as a flushing mechanism, increasing washout and consequently the recovery of the total tracer mass present in the system.

Although all scenarios presented high dispersion (*d* ≥ 1.21), indicating overall behavior with a tendency toward CSTR (Kadlec and Wallace 2009), a hydrodynamic evolution was observed over time. The dispersion index (*d*) decreased by 20% from Year 3 (NR‐3, *d* = 1.58) to Year 5 (average *d* = 1.26). The rainfall test (R‐5, *d* = 1.3) showed slightly higher dispersion than the dry‐weather test (NR‐5, *d* = 1.21), possibly due to turbulence induced by increased inflows. This reduction in dispersion over the years may be associated with system maturation, such as root formation and particle sedimentation (Jin et al. 2023; Miranda et al. 2019). As expected, the promotion of preferential pathways (short circuits and clogging) reduces dispersion (lowering *d*) and increases the number of tanks in series (raising *N*).

The TIS model (*N*) confirms this trend. The system evolved from *N* = 1.54 (NR‐3) to higher values in Year 5 (NR‐5: *N* = 1.83; R‐5: *N* = 2.07), an average increase of 26.7%. Therefore, both estimators (the decrease in *d* and the increase in *N*) reinforce the same conclusion: The VSSF‐CW is developing preferential pathways, showing a slight tendency to move away from the CSTR model (*N* = 1).

In full‐scale CWs, hydraulic behavior evolves over time because of root proliferation and the accumulation of biofilm and inorganic solids, all of which progressively modify pore structure and promote phenomena such as preferential flow, dead zones, and partial clogging. Superimposed on these internal changes, climatic drivers, including evapotranspiration and rainfall, alter hydraulic loading, residence time, and flow distribution. For this reason, CW design and operation must account for temporal variability by incorporating hydraulic safety margins, ensuring robust influent distribution, and implementing long‐term monitoring strategies capable of detecting shifts in flow patterns and emerging hydraulic constraints.

## 4 Conclusion

The tracer tests consistently showed that, across all three events (NR‐3, NR‐5, and R‐5), the system operates under a highly dispersive flow regime (*d* > 1.21; *N* < 2.07) with a tendency toward CSTR‐like behavior. In all cases, the wetland exhibited clear deviations from ideality, suggesting asymmetric flow paths and the occurrence of hydraulic short circuits. This recurrent pattern, independent of environmental or temporal conditions, confirms the presence of persistent internal flow heterogeneities.

A comparison between the NR‐3 and NR‐5 tests revealed significant differences (*p* < 0.05, *t* test) in the hydrodynamic parameters of the system. These results indicate that the wetland underwent measurable changes in its hydraulic behavior over the years, reflecting the effects of system maturation, root development, and structural evolution of the porous medium. Together, these findings demonstrate that long‐term operation progressively alters internal flow dynamics, with direct implications for pollutant–microorganism contact and overall treatment performance.

The R‐5 event significantly reduced rHRT and altered dispersion parameters (*p* < 0.05, *t* test), driven by dilution and flushing mechanisms that intensified advective transport. These results highlight the sensitivity of VSSF‐CWs to precipitation, particularly in regions with strong rainfall seasonality, and demonstrate the importance of incorporating climatic variability into hydrodynamic assessments.

The persistent preferential pathways and short circuits observed across all tests highlight the importance of inlet distribution systems capable of promoting more uniform flow, whereas the rainfall‐induced reductions in HRT underscore the need to consider storm events when sizing wetlands in regions with marked precipitation variability.

## Author Contributions

**Galilleu Silva:** investigation, writing – original draft, methodology, data curation. **Édio Damásio da Silva Júnior:** conceptualization, writing – review and editing, supervision.

## Conflicts of Interest

The authors declare no conflicts of interest.

## Data Availability

The data that support the findings of this study are available from the corresponding author upon reasonable request.
